# Advances in Deoxynivalenol Toxicity Mechanisms: The Brain as a Target

**DOI:** 10.3390/toxins4111120

**Published:** 2012-11-01

**Authors:** Marion S. Bonnet, Julien Roux, Lourdes Mounien, Michel Dallaporta, Jean-Denis Troadec

**Affiliations:** 1 Laboratory of Physiology and Pathophysiology of Somatomotor and Autonomic Nervous System, Faculty of Sciences and Technology, Escadrille Normandie-Niemen Avenue, Aix-Marseilles University, Marseilles 13397, France; Email: marion.bonnet@univ-amu.fr (M.S.B.); lourdes.mounien@univ-amu.fr (L.M.); michel.dallaporta@univ-amu.fr (M.D.); 2 Biomeostasis, Contract Research Organization, Faculty of Sciences and Technology, Escadrille Normandie-Niemen Avenue, Marseilles 13397, France; Email: julien.roux@biomeostasis.com

**Keywords:** mycotoxins, deoxynivalenol, hypothalamus, brainstem, anorexia, anapyrexia, cytokines, POMC, nesfatin-1

## Abstract

Deoxynivalenol (DON), mainly produced by *Fusarium *fungi, and also commonly called vomitoxin, is a trichothecene mycotoxin. It is one of the most abundant trichothecenes which contaminate cereals consumed by farm animals and humans. The extent of cereal contamination is strongly associated with rainfall and moisture at the time of flowering and with grain storage conditions. DON consumption may result in intoxication, the severity of which is dose-dependent and may lead to different symptoms including anorexia, vomiting, reduced weight gain, neuroendocrine changes, immunological effects, diarrhea, leukocytosis, hemorrhage or circulatory shock. During the last two decades, many studies have described DON toxicity using diverse animal species as a model. While the action of the toxin on peripheral organs and tissues is well documented, data illustrating its effect on the brain are significantly less abundant. Yet, DON is known to affect the central nervous system. Recent studies have provided new evidence and detail regarding the action of the toxin on the brain. The purpose of the present review is to summarize critical studies illustrating this central action of the toxin and to suggest research perspectives in this field.

## 1. Introduction

**Figure 1:**
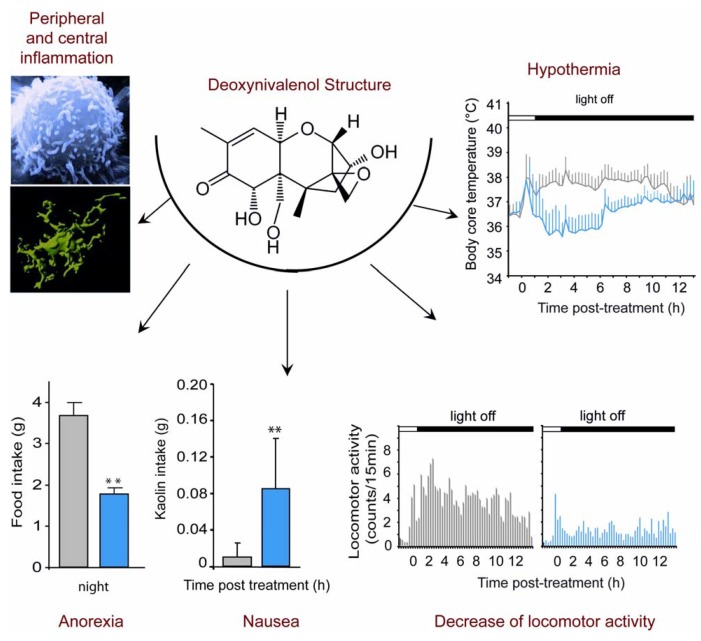
Symptoms associated with acute Deoxynivalenol (DON) intoxication. In addition to a peripheral and central inflammation, when administered *per os* at the end of the light phase at a dose of 12.5 mg/kg, DON induces a decrease in food intake observed overnight following the treatment, and an increase in Kaolin intake measured 24 h after administration suggesting a gastric discomfort. Moreover, telemetric analyses show a 2 °C decrease of body core temperature and a locomotor activity decrease during the night in DON-treated animals compared with control animals.

Deoxynivalenol (DON) is a secondary metabolite mycotoxin produced more especially by *Fusarium graminearum* and *culmorum*. These fungi can grow on various cereals such as wheat, barley, oat, rye, maize and rice. The extent of cereal contamination is strongly associated with rainfall and moisture at the time of flowering and with grain storage conditions. Deoxynivalenol represents the major fungus metabolite which can be found on feed grains. The chemical name of DON is DON 12,13-epoxy-3α,7α,15-trihydroxytrichothec-9-en-8-on, (C_15_H_20_O_6_); it has a polar organic structure and a molecular weight of 296.36 g/mol. The keton position in C_8_ is a feature of the class B trichotecenes [1]. Due to its high melting point (151 °C–153 °C), DON withstands cereal processing and cooking and thus constitutes an important food contaminant [2,3,4,5]. The consumption of DON-contaminated food induces mycotoxicoses in farm animals as well as in humans. DON toxicity is characterized by a set of symptoms including diarrhea, vomiting, anorexia, reduced weight gain, neuroendocrine and immunological changes, leukocytosis, hemorrhage, circulatory shock, and can ultimately lead to death (Figure 1). Its capacity to induce vomiting episodes in various species including humans explains its commonly used nickname “vomitoxin” [6]. During the last two decades, many studies have described DON toxicity using diverse species as a model, where DON consumption was shown to affect numerous physiological functions such as food intake, reproduction or immunity. While the toxin action on peripheral tissues and organs is well documented, studies aiming to identify the impact of DON on the brain are relatively less abundant. Based on evidence showing that DON ingestion could disrupt brain neurochemistry, recent studies have brought new information regarding its central effects especially in relation to anorexia/nausea. The purpose of the present review is to summarize recent critical studies illustrating the central action of the toxin and to propose future research directions in this field. 

## 2. DON, Food Refusal, Emesis and Anorexia

In humans, mycotoxicosis observed after consumption of feed contaminated with DON, leads to vomiting, reduced food intake, abdominal pain and diarrhea (for review [7]). It is well established that DON consumption also results in decreased weight gain and food intake and altered nutritional efficiency in different models including poultry, rodents and pigs. The key studies evaluating emesis, growth and food intake in various species and in response to different DON doses were gathered by Pestka and Somlinski [8]. The intensity of the effects depends on the dose, the species, the duration of consumption, DON purity and the route of administration. For instance, 50 to 100 µg/kg orally administered DON induced vomiting in swine [9,10,11]. This emetic effect can also be produced by ingestion of feed contaminated with DON at a dose of 20 ppm (equivalent to 0.15 mg/kg BW/day; [12]. The ingestion of naturally contaminated food with 1 to 2 ppm DON caused partial feed refusal, whereas 12 ppm caused complete refusal [13,14]. In mice*, *consumption of 2 ppm purified DON during 8 weeks (equivalent to 0.3 mg/kg BW/day) provoked decreased growth and 0.5 ppm (equivalent to 0.075 mg/kg) reduced food intake [15]). A drastic decrease in food intake and a related loss of weight was observed in mice consuming a diet containing 2.5 ppm purified DON (equivalent to 0.36 mg/kg) during one week [16]. Similarly, in the rat, the consumption of 0.5 mg/kg purified DON during 9 weeks resulted in growth reduction and decreased food intake [17]. Similar observations have been made in dogs and cats fed for 2 weeks with diet naturally contaminated with 6 to 8 ppm DON (equivalent to 0.4 mg/kg) [18]. 

## 3. DON-Induced Anorexia in Mice Models

Thus, although it is clear that DON ingestion induces a reduction of food intake in different animals models, the feature of this DON-induced anorexia remains poorly characterized. Recently, a noticeable effort was made to describe more precisely the DON-induced anorexia using mice as a model [19,20,21,22,23]. While swine was reported to be the most sensitive species to DON, numerous *in vivo* and *in vitro* DON toxicity studies were conducted on rodents and especially on mice which can be considered as a good model for the study of DON toxicity. In a model of high fat diet-induced obesity (HFD; 60% kcal from fat), mice consuming 5 or 10 ppm DON for 10 weeks exhibited a clear reduction of weight gain [19]. In another set of data originating from the same group using HFD-induced obesity (54% and 60% kcal from fat), DON consumption (equivalent to 10 mg/kg) decreased body weight, fat mass and food intake. These physiological modifications were associated with reduced plasma insulin, leptin, insulin-like growth factor 1, and an increase in hypothalamic mRNA level of orexigenic agouti-related protein [23]. Flannery and collaborators [20] measured noctural food intake on mice fasted during light cycle and treated with i.p. 1 and 2.5 mg/kg or oral gavage 0.5 to 5 mg/kg DON. Food intake was measured 2 h and 16 h after toxin exposure. These authors report that DON caused a rapid feed refusal that was evident 2 h after toxin administration for the highest DON doses. Interestingly, this effect was dose-dependent and transient, since 16 h after DON-administration, an increase in food intake occurred in DON-treated mice when compared with vehicle-treated animals. Using a different experimental paradigm, we recently obtained additional information regarding DON-induced anorexia [21,22]. In these studies, we performed acute *per os* DON administration (6.25, 12.5 and 25 mg/kg) at the end of the light phase and monitored noctural food intake in mice fed* ad libitum*. In this context, *per os* intoxication induced a dose-dependent reduction in daily food intake by especially decreasing night-time food consumption in animals fed *ad libitum *(Figure 1). Anorexia was observable as soon as 3 h after toxin exposure for 12.5 and 25 mg/kg and 6 h for the lowest dose. This anorexigenic effect lasted up to 24 h with 6.25 and 12.5 mg/kg DON and on over 72 h with the highest dose. When performing a meal pattern analysis, which provides an accurate description of the eating behavior through a continuous and automated recording of food intake, we revealed a DON-induced reduction in meal frequency (satiety) and meal size (satiation) by 44.2% and 68% respectively. In accordance, the inter-meal interval increased by 68% in the presence of the toxin. The effect on meal frequency was evocative of nausea-induced anorexia as revealed by studies showing that meal frequency is especially affected by toxicological compounds such as lithium and lipopolysaccarids (LPS; [24,25]). In rodents, emesis cannot occur but DON can induce nausea. Indeed, we measured DON-induced nausea in mice by measuring kaolin consumption, a non-nutritive substance, the intake of which serves to evaluated nausea in rodents [26]. The results showed that DON at 12.5 mg/kg induces a significant increase in kaolin intake for the 24h following toxin administration (Figure 1). This result suggests a nauseous effect of DON in mice. However, kaolin intake was poorly correlated with standard chow intake, suggesting that nausea does not totally explain feed refusal. On the other hand, the consequence of DON treatment on meal size supports the view that DON acts on meal termination mechanisms. A reduction in meal size but not in meal frequency is typically observed with physiological inhibitors of food intake such as leptin [27], cholecystokinin [28], peptide YY 3-36 [29], insulin [30]. Hence, taken together these results reveal a complex action of DON on food intake where DON-induced anorexia depends not only on modification of satiety but also of satiation. In parallel to food intake a modulation of body temperature and locomotor activity was also recorded in DON-treated mice (Figure 1). This set of symptoms *i.e.*, anorexia/nausea, reduction of locomotor activity and modulation of body core temperature is evocative of sickness behavior occurring in response to inflammatory challenges (for review, [31]). 

## 4. Sickness Behavior and Central Cytokines Expression

The survival of a pluricellular organism depends on its capacity to fight against infections and to store energy. The immune and metabolic systems, among the most essential ones of the animal kingdom, are closely linked and interdependent. Numerous hormones, cytokines, or bioactive lipids can exert metabolic and immune functions. Inflammation is a response coordinated by an organism that allows it to struggle against an attack. This reaction leads to deep physiological and behavioral changes due to the activation of the innate immune system and the recognition of molecular motives linked to the pathogen. The classical inflammatory response called “acute phase reaction” implicates metabolic modifications, redistribution of energy and the use of lipid supplies. These modifications require redefining physiological preferences for an efficient adaptation of organism with the intervention of the central nervous system (CNS) which coordinates the installation of the central element of the acute phase reaction. This central component of the acute phase reaction consists of deep behavioral changes called “sickness behavior” which includes fever, activation of the stress axis and reduction of food intake. In this context during an infection and inflammation, pro-inflammatory cytokines produced by a variety of cells reach and interact with brain regions that control ingestion *i.e.*, the hypothalamus and the dorsal vagal complex (DVC) to induce anorexia. Peripheral immune signals reach the brain via a humoral communication through circumventricular organs such as the anteroventral region of the third ventricle and the area postrema (AP) and via a neuro-immune gut-brain pathway that mainly involves the sensory vagal afferent innervation (see for review [32]). Accordingly, anorexic doses of peripheral cytokines such as IL-1β activate the primary projection area of the vagus nerve which is located in the DVC. In turn, multiple secondary projection sites of the vagus nerve involved in the processing of gut visceral information and in the control of food intake *i.e.*, the hypothalamus, the central nucleus of the amygdala and the bed nucleus of the stria terminalis, are activated [33]. Alternatively, in response to peripheral inflammation, the brain can also be a source of cytokines, as demonstrated by several studies [34]. Using genetic models exhibiting impairment of LPS and IL-1β signaling, these studies have shown that this *de novo* central production of cytokines is required to obtain a sustained anorexia in response to LPS or IL-1β administration [35,36]. Finally, a modulation in the expression of neurotransmitters and anorexigenic or orexigenic neuropeptides in the hypothalamus was proposed to contribute to the anorexic behavior induced by inflammatory signals (for review [32]). 

## 5. DON, Cytokines Expression and Anorexia

In the mid-nineties, Azcona-Olivera and colleagues [37] have reported that an oral exposure to 5 and 25 mg/kg BW of DON results in mice in an increased cytokine production in spleen, Peyer’s patches, liver, kidney and small intestine. Using RT-PCR and Southern-blot, the authors demonstrated the increased abundance of IL-1 β, IL-6, TNF-α and to a lesser extend TGF-β and INF-γ mRNA shortly after DON exposition (2 and 4 h). This pioneer study was confirmed and strengthened by numerous other works performed on different models including mice, pigs or human cell lines [38,39,40,41,42]. For instance, acute oral exposure of B6C3F1 mice to DON (5 and 25 mg/kg BW) was shown to increase, in spleen and Peyer’s patches, the expression of INF-γ, IL-2, IL-4 and IL-10 in addition to the pro-inflammatory cytokines mentioned above [42]. In the same way, piglets intravenously injected with 1 mg/kg BW DON exhibited a modulation of IL-1 β, IL-6 and TNF-α expression in lymphoid organs [39]. In human macrophages, 100 to 1000 ng/mL of DON and other 8-ketotrichothecenes significantly upregulated the expression of TNF-α, IL-6 and IL-8 [43]. In Jurkat T-cell, DON (32.5–500 ng/mL) upregulates IL-2 and IL-8 production [44]. Finally, in human monocyte cell line, 1 µg/mL DON stimulated IL-8 mRNA and protein production [45]. Interestingly, several studies reported the amplified proinflammatory cytokine induction during cotreatment with both DON and bacterial LPS [46,47,48]. Given the well-known anorexigenic effect of proinflammatory cytokines, their increased expression observed in response to DON intoxication was proposed to drive associated symptoms including anorexia and reduced weight gain [49]. Supporting this hypothesis, we have recently shown that *per os* DON administration induced an up-regulation of IL-1β, IL-6 and TNF-α within two central structures *i.e.*, hypothalamus and DVC [22]. These results constituted the first demonstration that *per os* DON administration results in central neuroinflammation. As mentioned above, these structures are involved in the regulation of food intake. Altogether, these data strongly suggest that peripheral and also central-borne cytokines may participate in the onset of reduced food intake observed in response to DON intoxication. Although attractive and plausible, this hypothesis remains to be experimentally demonstrated. Even though inflammation-related effectors are strongly expressed during DON intoxication, the arguments supporting their triggering role in DON-induced anorexia are still limited. First, TNF-α receptors KO and IL-6 KO mice do not exhibit any reduced susceptibility to DON-induced anorexia [50,51]. Prostaglandins (PGs) are key inflammatory mediators acting downstream of proinflammatory cytokines which induce some of the symptoms of sickness behavior such as anorexia ([52]). PGH2, the end product of cyclooxygenase (COX) enzymes, can be converted into various eicosanoids such as PGD2, PGF2α, PGE2, prostacyclin and thromboxane A2 [53], each compound having their own specific biological activities. PGE2 are the most potent PG in inducing anorexia when centrally administered [54]. The microsomal PGE synthase-1 (m-PGES-1) belongs to the MAPEG (Membrane Associated Proteins in Eicosanoid and Glutathione Metabolism) superfamily and catalyses the final step of PGE2 synthesis [55]. mPGES-1 has been described as an inducible enzyme which expression is stimulated by pro-inflammatory agents [56,57,58] and silencing mPGES-1 gene prevents IL-1 induced anorexia [59]. We recently reported the increased expression of COX-2 and mPGES-1 transcripts within the hypothalamus and brainstem of DON-treated mice [22]. Different transgenic mouse models were used to investigate the involvement of PGs in DON-induced anorexia and reduced weight gain. COX-2 knock-out (KO) mice were reported to exhibit a decrease in body weight comparable to that of wild type littermate when exposed to chronic consumption of DON during 16 weeks [60]. Moreover, silencing PGE2 signaling pathways using mPGES-1 KO mice did not modify the anorexigenic response to DON [22]. 

**Figure 2:**
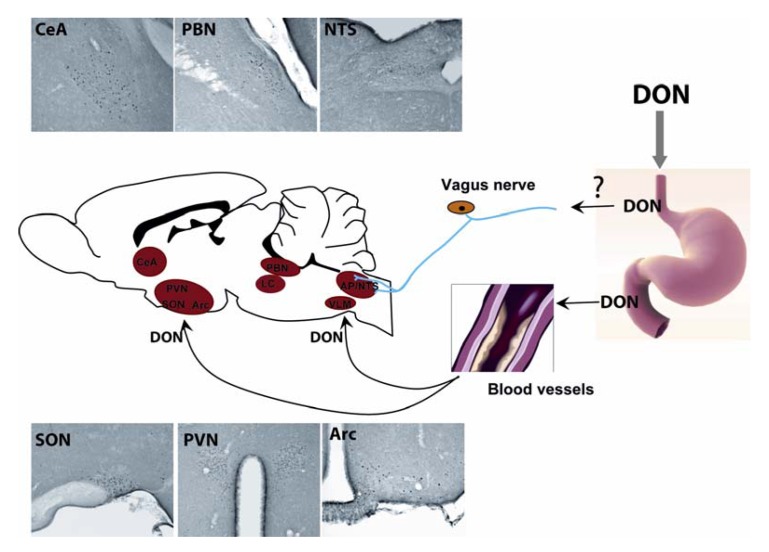
Central structures activated by acute DON intoxication. *Per os* administration of DON induces an increase in the number of c-Fos positive neurons in several brain nuclei such as the hypothalamus (paraventricular nucleus, arcuate nucleus, supraoptic nucleus), the dorsal vagal complexe (DVC) at the brainstem level (nucleus tractus solitarius, dorsal motor nucleus of the vagus), the pons (parabrachial nucleus, locus coeruleus) and the central amygdala. During an oral intoxication, DON may be conveyed to the brain where it may activate these structures, leading to the establishment of symptoms.

## 6. Brain Structures Activated during Acute DON Intoxication

For many years, the expression of the c-fos gene or protein has been considered as a high resolution marker of neuronal activity since c-Fos protein is expressed in neurons whose activity is strongly stimulated by synaptic input [61,62]. Even if one must bear in mind that not all activated neurons show c-Fos induction, and that the threshold of c-Fos protein induction may differ between subpopulations of CNS neurons, the use of c-Fos expression remains a useful approach to identify and investigate neuronal groups activated in response to different challenges throughout the brain. We recently used this approach to identify central structures activated during DON intoxication [21,22]. We revealed the activation of key autonomic areas, hypothalamic nuclei and parts of the amygdala. This c-Fos analysis was performed three hours after peripheral DON administration. At this time point, activated structures could be considered instrumental in DON-induced responses as anorexia and associated symptoms were ongoing. c-Fos immunoreactivity was observed in key autonomic regulatory nuclear groups, including the nucleus tractus solitarius (NTS), area postrema (AP), ventrolateral medulla (VLM), lateral parabrachial nucleus (LPB), locus coeruleus (LC). At the hypothalamic level, a significant increase in c-Fos immunoreactivity was mostly observed in the paraventricular hypothalamic nucleus (PVN), arcuate nucleus (ARC), median eminence (ME), and supraoptic nucleus (SON). The central nucleus of the amygdala (CeA) and the dorsolateral division of the bed nucleus of the stria terminalis (BST) which are involved in the integration of emotional stimuli, also displayed a strong c-Fos signal in DON-challenged mice (Figure 2). Noticeably, the serotoninergic raphe formation did not exhibit any DON-induced c-Fos expression whatever the rostro-caudal level considered [22]. Interestingly, this pattern of c-Fos distribution closely resembles c-Fos immunoreactivity described in previous studies performed to identify neurocircuitry involved in the coordinated autonomic, endocrine, and behavioral response to immune challenge [63,64,65,66]. The strong c-Fos induction in structures strongly involved in food intake regulation *i.e.*, the NTS, Arc, and PVN, can be linked to the DON-induced reduction in food intake. Moreover, the c-Fos induction within the brainstem and particularly the AP was evocative of nausea. Using systemic DON administration in rats, Ossenkopp and colleagues [67] have indeed reported the induction of conditioned taste aversion, which was mediated by the AP. The strong increase in c-Fos immunoreactivity in specific regions of the hypothalamus including the preoptic area/anterior hypothalamus [68], the PVN and SON, together with the activation of LC and NTS [69] could also explain the decreased body temperature observed in response to DON [22]. These novel results, while interesting and informative, remain incomplete. Actually, only one DON dose *i.e.*, 12.5 mg/kg was tested and the c-Fos activation pattern was assessed at a single time-point *i.e.*, 3 h after treatment. Future studies should evaluate the impact of lower doses of toxin and a time-course of brain activation to more precisely understand the impact of DON on various brain regions. 

## 7. Brain Neurochemistry and DON

The effect of DON on brain neurochemistry has been investigated in different species by several authors for many years [11,70,71,72,73,74,75]. It was hypothesized that alterations of brain neurochemistry might be one of the possible mechanisms underlying *Fusarium* mycotoxin-induced feed refusal, emesis and anorexia. In 1988, Fitzpatrick and colleagues [76] analyzed brain biogenic monoanines (serotonin, 5-HT; 5-hydroxyindole-3-acetic acid, 5HIAA; norepinephrine, NE ; dopamine, DA) in both rats and chickens at different time (2 to 48 h) after an oral dose of 2.5 mg/kg DON. In the rat, while no difference was observed when the whole brain was considered, 5-HT and 5-HIAA were elevated in all brain regions studied including pons and medulla oblongata, cerebellum, hypothalamus, hippocampus and cortex. These alterations of the serotoninergic system were not observed in poultry, where NE and DA decreased in the hypothalamus, hippocampus and pons. Following acute DON administration (0.25 mg/kg intravenous) in swine, modifications of brain amine levels were also detected during the 24 h post-intoxication [73]. More precisely, DON administration was reported to increase NE and decrease DA concentrations in the hypothalamus, frontal cortex and cerebellum while 5-HT content, which initially increased in the hypothalamus (1 h post-treatment), diminished in the hypothalamus and the cortex at 8 h. Likewise, intravenous (10 µg/kg) and *per os* (30 µg/kg) DON administrations were shown to increase 5-HIAA in the cerebral spinal fluid, suggesting an increased CNS serotoninergic activity [72]. More recently, the effects of feeding (21 days) with a blend of grains naturally contaminated with *Fusarium* mycotoxins (containing 5 ppm DON, 0.5 ppm 15-acetylDON and 0.4 ppm zearalenone) on brain regional neurochemistry of starter pigs and chickens were studied [74,75]. In pigs, contaminated food intake also results in modifications of brain bioamine contents. Mainly, this study reports an increased concentration of 5-HT within the cortex and a decreased hypothalamic tryptophan concentration. Moreover, hypothalamic NE and pons DA concentrations decreased after inclusion of contaminated grains in the diet. In broiler chickens, feeding with contaminated grains induced an increase of 5-HT, 5-HIAA concentrations within the pons and cortex, and an increase in NE and DA concentrations in the pons [74,75]. Finally, one study was recently performed with turkeys fed with grains contaminated with *Fusarium* mycotoxins (equivalent to 2.2 and 3.3 mg DON per kg of feed) during 6 weeks [70]. In this model, 5-HIAA concentration and 5-HIAA: 5-HT ratio decreased in the pons, but no significant effects on 5-HT, 5-HIAA, NE and DA concentrations were observed in the hypothalamus and cortex. Despite the use of different intoxication models (DON doses, routes of administration) and apparent interspecies dissimilarities, these alterations in bioamine turnover, and particularly in the serotoninergic system, were proposed to play a role in the triggering of feeding refusal, emesis and anorexia observed during DON intoxication [7]. 5-HT has well-known emesis and food intake reducing effects [71]. In accordance, 5-HT receptor antagonists, *i.e.*, 5-HT3 and to a lesser extend 5-HT2, significantly prevented DON-induced vomiting (intravenous: 80 µg DON per kg BW or oral: 300 µg DON per kg BW; [11]). To discriminate between peripheral and central serotoninergic systems in DON-induced vomiting and anorexia, Prelusky [77] studied the effect of intravenous or intragastric DON administration on plasma concentrations of 5-HT and related metabolites in pigs. Interestingly, at doses able to evoke emesis, the toxin did not induce any change in blood monoamine levels, suggesting that the increased activity of the central serotoninergic system may account for the altered feeding behavior and emesis observed during DON intoxication. It should be noted that using an *in vitro* membrane receptor binding assay, a functional interaction of the toxin with serotoninergic receptors was ruled out [78]. Nevertheless, whether these alterations of brain neurochemistry are responsible for the DON-induced anorexia remains unknown. In a recent study using c-Fos expression as a marker of neuronal activation and performed on mice acutely intoxicated with 12.5 mg/kg DON, we observed an activation of the A1/C1 and A2/C2 catecholaminergic groups [21], which was evocative of the modulation of NE concentrations observed after DON intoxication in different models [11,70,73,74,75,76]. The activation of these catecholaminergic groups by DON also demonstrates the activation of brainstem/hypothalamus connecting networks. On the other hand, we did not observe any activation of the serotoninergic raphe nucleus in this model. This result is quite surprising since the modulation of brain 5-HT concentrations in intoxicated animals has been reported (see above). The specie used *i.e.*, mice and the time point studied in this work *i.e.*, 3 h after intoxication, may explain the discrepancies between our results and previous data. To clarify this point, more complete analyses need to be done in mice but also in other species where monoamine dosages during DON-intoxication are available, e.g., pigs. 

## 8. Feeding Circuits and DON

In order to shed light on the mechanisms involved in DON-induced anorexia and identify cell populations implicated, we have recently searched for the possible activation of central pathways strongly dedicated to the control of food intake after administration of the toxin. In this context, we have reported that a significant proportion of POMC- and nesfatin-1-expressing neurons located both in the hypothalamus and NTS were activated during DON intoxication [21]. Both POMC and nesfatin-1 neurons are strongly involved in the reduction of food intake under physiological condition. POMC-expressing neurons also called “first-order neurons” are well recognized as the major component in the control of energy homeostasis both in rodents and humans [79]. POMC neurons which relay the anorexigenic action of leptin and insulin, release the α-melanocyte stimulating hormones (αMSH). This hormone acts on melanocortin 3 and melanocortin 4 receptors (MC3R and MC4R) of neurons mainly located in the PVN to reduce appetite. Both insulin and leptin increase the activity of anorexigenic POMC neurons in the ARC. Thus, the increase activity of POMC neuron induces a decrease in food intake and an increase in energy expenditure. Similarly, nesfatin-1, an 82 amino-acid peptide, was identified in 2006 by Oh-I and colleagues [80]. This peptide exerts potent anorexigenic action after either peripheral [81] or central administration [80]. In addition to its expression by peripheral tissues *i.e.*, the stomach, pancreas and adipose tissues [82], nesfatin-1 was also found to be expressed in neurons of various brain areas including hypothalamic (PVN, ARC) and brainstem nuclei (NTS, DMNX) [83]. Nesfatinergic neurons located in these nuclei are activated in response to refeeding or intraperitoneal injection of the anorexigenic hormone cholecystokinin indicating that central nesfatin-1 could participate in the meal termination mechanisms [84,85]. Moreover, nesfatin-1 has a food intake-reducing effect that is linked to the recruitment of the melanocortinergic pathway [80]. The activation of DON-induced POMC and nesfatin-1 expressing neurons in mice suggests that the release of these anorexigenic and related compounds could partake in the anorexigenic action of the toxin. Strengthening these results, we observed that DON modulated mRNA levels of the hypothalamic melanocortinergic system including POMC, CART and MC4R, whereas mRNA of orexigenic effectors *i.e.*, NPY and AgRP remained unaffected. Altogether, these studies provide strong additional support to the view that DON directly or indirectly interferes with neuronal networks devoted to central energy balance and that this action could partly explain the DON-induced hypophagia observed in response to the toxin. 

## 9. DON May Target the Brain

Although DON effects on brain structures and neurochemistry were clearly established by a body of evidence, little is known about the routes used by the toxin to signal from the periphery (gastrointestinal tract, blood) to the brain. The vagal nerve which conveys information from the visceral tract to the brainstem is a good candidate to explain the impact of the toxin on the brain. Unfortunately, very few data are available regarding this hypothesis. In a recent study, we have shown that unilateral cervical vagotomy did not modify the c-Fos activation in the brainstem induced by *per os* 12.5 mg/kg DON [21,22]. This result seems to rule out the involvement of the vagus nerve, at least with the dose employed. Nevertheless, additional experiments with other DON doses are clearly needed to definitively settle this point. Alternatively, it is conceivable that the toxin enters the brain to directly modify neuronal function. In accordance, following i.v. toxin administration, DON is rapidly detected (less than 2.5 min) in the cerebral spinal fluid of swine and sheep demonstrating that this toxin reaches the brain [86]). DON concentrations within the brain reach a maximum after 5–10 min in sheep and 30–60 min in swine. In the same way, DON was detected in hen or mouse brains after acute or chronic (6 days) DON administration [49,87]. Using radiolabelled-DON or ELISA DON detection, these authors detected the toxin within the brain of these species following *per os* administration. Interestingly, after oral administration of 25 mg/kg DON in mice, the toxin concentrations detected in the brain were lower (~1 µg/g) than in other tissues *i.e.*, spleen (~7.3), liver (~19.5), heart (~6.8), and kidney (~7.6), probably due to the low permeability of the BBB to the toxin. Of course the toxin could also infiltrate the brain through the circumventricular organs. In support of this assumption, DON was shown to induce c-Fos staining in circumventricular organs (AP and ME) and surrounding structures (NTS and ARC; [21,22]). Moreover, using systemic DON administration in rats, Ossenkopp and colleagues [67] reported that area postrema ablation prevents DON-induced conditioned taste aversion. Finally, to assess the direct action of the toxin on the brain, we recently performed the first i.c.v. (lateral ventricle) DON administration in the mouse (2–20 µg/g; [21]). The doses used in these i.c.v. experiments were compatible with DON brain levels found after *per os* administration (25 mg/kg; [38]), but totally inefficient to modify feeding behavior when peripherally administered. In these conditions, DON dose-dependently decreased meal frequency and size and augmented intermeal intervals in the same way as after *per os* administration (12.5 mg/kg DON). Like *per os* administration, DON central injections also result in a modification of body core temperature and locomotor activity. DON-induced c-Fos expression pattern following i.c.v. and *per os* DON administrations were also similar. Lastly, POMC and nesfatin-1 positive neurons of both the NTS and hypothalamus were found activated after i.c.v. DON injections to a same extent as after *per os* DON administration. Taken together, these results suggest that DON might directly target the brain to modify feeding behavior acting on anorexigenic neurocircuitry. 

## 10. Perspectives

Despite significant progress in the identification of neuronal circuitry and neuropeptides recruited during DON intoxication, the precise physiological mechanisms and cellular pathways underlying the central action of the toxin remain mostly unidentified. The hypothesis of the instrumental role of cytokines in the triggering of DON-induced anorexia, while attractive, remains to be established. To date, genetic models displaying an altered inflammatory response, *i.e.*, IL6 KO [51], TNFR KO [50], COX-2 KO [60] and mPGES-1 KO [22], failed to confirm the involvement of inflammatory signals in the reduction of feeding behavior induced by the toxin. Surprisingly, the outcome of lessening IL-1β signaling on DON-induced anorexia has not been tested to date. Yet among the inflammatory cytokines, IL-1β seems to be the most potent in inducing sickness behavior, since its peripheral or central injection reproduces the set of non-specific symptoms of inflammation including decreased motor activity, social withdrawal, anorexia and fever (for review [88]). Accordingly, strategies aiming to limit its action have been shown to attenuate most of the symptoms observed during infection and inflammation [89,90,91]. So in the future, it should be of interest to test the impact of interleukin-1 receptor antagonist (IL-1Ra) on DON-induced anorexia, or to evaluate the hypophagic response in interleukin-1β-deficient mice. Alternatively, important information could result from the use of mice exhibiting an invalidation of the intracellular protein MyD88, the central adapter protein mediating activation signals emanating from the IL-1 receptor [92]. As mentioned above, we recently demonstrated the activation of POMC-expressing neurons in both the hypothalamus and brainstem [21] and the modulation of POMC expression in the hypothalamus during acute DON intoxication. To test the hypothesis of melanocortinergic pathway involvement in DON-induced hypophagia, the central injection of synthetic MC3R/MC4R antagonists ought to be tested in different models of DON-intoxication. This point is crucial since nesfatin-1 has a food intake-reducing effect that is also linked to the recruitment of the melanocortinergic pathway [80]. Another intriguing question regarding the central action of the toxin is the pattern of activated structures observed after *per os* DON administration. Surprisingly, DON does not induce a neuronal activation throughout the brain, but on the contrary the number of central structures found activated in response to gastric administration of the toxin are quite limited [21,22]. What is the mechanism underlying this specificity? We believe that the proximity to circumventricular organs *i.e.*, AP and median eminence, which facilitates the toxin penetration into the brain could explain the strong activation in adjacent structures and connected nuclei such as the NTS, Arc or PVN. This hypothesis should be confirmed in the future. Nevertheless, this does not make clear why only anorexigenic pathways are modulated in these structures [21].

The cellular mechanisms through which DON could modify neuronal activity and brain neurochemistry are also still unknown. DON binds to ribosomes and in turn inhibits protein synthesis (for review [7]). In mice, *per os* DON administration (5 to 25 mg/kg) inhibits protein synthesis in the spleen, peyer’s patches, kidney, liver, intestine and plasma from 3 h to 9 h post-treatment [37]. It was recently proposed that the ribotoxic effect of the toxin induces a modulation of mitogen activated protein kinases (MAPKs) activity. In turn, MAPKs activation may result in the activation of transcription factors that induce for instance cytokine or COX-2 expression [93,94,95]. It was proposed that other signaling factors such as double stranded RNA-activated protein kinase or hematopoietic cell kinase relay DON toxicity from ribosome inhibition to MAPKs activation [96,97,98]. Whether such mechanisms intervene in the brain and may account for the neuronal activation observed in response to orally administered DON remain to be evaluated. Alternatively, other mechanisms were proposed to explain trichothecene toxicity especially T-2 toxin [99,100]. On L-6 myoblasts, T-2 toxin has multiple effects on cell membrane functions independently of protein synthesis inhibition. T-2 Toxin modifies the activity of amino acid and glucose transporters as well as calcium and potassium channel activities [99]. This toxin was also shown to increase cellular calcium concentration in the human promyelotic line HL-60 [100]. The assumption that DON induces such modulations of membrane functions or intracellular pathways remains to be evaluated. Since DON was shown to induce rapid behavioral responses (1 h), *i.e.*, anorexia, anapyrexia, reduced locomotor activity, the possibility of an action independent of protein synthesis inhibition can be reasonably envisaged. In neurons, such alterations can result in modifications of neuronal electrical activity. Electrophysiological experiments performed on brain slices originating from DON-administered animals or on hypothalamic or brainstem slices acutely treated with the toxin could bring essential information regarding the central action of the toxin. 

## 11. Conclusions

From pioneer studies and more recent works, it clearly appears that DON acts at the brain level to modify neurochemistry and neuronal activity. In turn, behaviors regulated by the central nervous system are modified. Notwithstanding these interesting progresses, the study of DON impact on brain functioning is still in its infancy. Among the different aspects that must be addressed in this field in the close future, the study of the possible consequences of chronic DON consumption on long-term brain deregulation will surely constitute a major issue and a great challenge.
